# Assessment of lesbian, gay, bisexual, transgender, and questioning experiences within a large southeast training program

**DOI:** 10.1080/10872981.2022.2093692

**Published:** 2022-06-30

**Authors:** Zachary W. Walker, Mary Appah, Inmaculada Aban, Brenessa M. Lindeman, Latesha E. Elopre, Alice R. Goepfert, Samantha V. Hill

**Affiliations:** a Department of Obstetrics & Gynecology, University of Alabama at Birmingham, Birmingham, AL, USA; b Department of Biostatistics, University of Alabama at Birmingham, Birmingham, AL, USA; c Department of Surgery, University of Alabama at Birmingham, Birmingham, AL, USA; d Department of Medicine, University of Alabama at Birmingham, Birmingham, AL, USA; e Department of Pediatrics, University of Alabama at Birmingham, Birmingham, AL, USA

**Keywords:** LGBTQ, physician, awareness, environment

## Abstract

The USA has become increasingly diverse resulting in greater strides to improve workforce diversity and inclusivity. The objective of this study is to compare the experiences of trainees in Graduate Medical Education who identify as Lesbian, Gay, Bisexual, Transgender or Questioning (LGBTQ) to the experiences of non-LGBTQ trainees within the medical workplace. We conducted a cross-sectional, exploratory survey from 1 December 2020 to 14 January 2021 at a single, large teaching institution. We collected data anonymously and stored it in a REDCap database. We excluded surveys in which trainees did not respond to sexual orientation. We used contingency tables and Fisher’s exact test to identify outcomes associated with sexual orientation and gender identity particularly with regard to professionalism, well-being, and satisfaction with training. We distributed the survey to 840 trainees. 730 trainees were included (23 (3.2%) LGBTQ and 707 (96%) Straight). LGBTQ trainees were more likely to experience offensive remarks based on race/ethnicity (p = 0.03) and sexual orientation (p = 0.01). Secondary analysis based on race found that Blacks and Other were more likely to report differences based on professionalism and satisfaction with their training program. There was no difference seen among LGBTQ trainees based on race. We found trainees who identified as LGBTQ were more likely to experience discrimination/microaggressions. Also, racial and ethnic groups that are underrepresented in medicine were more likely to encounter discrimination and dissatisfaction with their training. More efforts are needed in academics to promote safe and supportive LGBTQ and minority training experiences.

## Introduction

The USA has become increasingly diverse in relation to race, ethnicity, and sexual and gender identity resulting in greater strides to improve workforce diversity and inclusivity. In 2009, the Liaison Committee on Medical Education and the Association of American Medical Colleges (AAMC) created formal recommendations to promote the accessibility of medical education to diverse applicants and improve representation of racial and ethnic groups that are underrepresented in medicine relative to their numbers in the general population [1,2]. Approximately 3.5% of Americans identify as lesbian, gay, or bisexual; and approximately 0.3% identify as transgender[3]. However, it is currently unclear how many healthcare physicians and trainees identify as lesbian, gay, bisexual, transgender, and questioning (LGBTQ) making it difficult to adequately quantify optimal diversity among Graduate Medical Education (GME) trainees regarding sexual orientation and gender identity and better reflect national demographics as well as patient population needs.

Keeping with the AAMC guidelines, LGBTQ trainees must be afforded the opportunity to learn and practice in welcoming environments that are responsive to their professional needs. However, members of the LGBTQ community working in healthcare are faced with numerous challenges, such as lack of mentorship, fear of discrimination/harassment, and lack of promotion within the workforce [4,5]. One could even go further to say that LGBTQ trainees are suffering through their postgraduate training in these environments in hopes of achieving their dreams of becoming physicians. Due to these circumstances, it is also difficult for academic institutions to retain LGBTQ residents, fellows, medical students, and faculty, especially those who may not be “open’ about their sexual orientation or gender identity. Furthermore, LGBTQ physicians and trainees may experience diminished interest in academic careers and report lower career satisfaction as faculty due to discriminatory and negative experiences during their training [4,5]. In addition, LGBTQ individuals who conceal their identity have increased rates of depression and anxiety which ultimately translates to an inability to focus on their academics or their clinical performance [6,7]. Thus, the lack of an inclusive environment in the workplace leads to an overall poor well-being of LGBTQ trainees and physicians.

In Alabama, approximately 3.1% of adults identify as LGBTQ[8]. The current prevalence of LGBTQ trainees and physicians, as well as their well-being, is unknown at the largest academic medical center in Alabama because there is not a standardized, routine way of collecting sexual orientation and gender identity data for GME trainees. Insights gained from such an evaluation can also be applied to other university-affiliated hospitals complying with AAMC guidelines to improve diversity and working environments for LGBTQ trainees. The site of this study is an active participant in the Healthcare Equality Index (HEI) survey which is a ‘benchmark tool that evaluates healthcare facilities’ policies and practices related to the equity and inclusion of their LGBTQ patients, visitors and employees’[9]. The study site is recognized as a 2020 LGBTQ Healthcare Equality Leader. However, there is no benchmark to represent the equality of LGBTQ trainees.

The principal theory behind exploration of this environment is the Positive emotion, Engagement, Relationships, Meaning, and Accomplishment (PERMA) theory of well-being [10]. The first element of the PERMA theory is positive emotion. Positive emotion is the most direct path to well-being in the sense that a positive mindset is associated with being happy. The second element is engagement. Engagement refers to doing something that one can be engrossed or absorbed in that ultimately brings them happiness. The third element is relationships which is self-explanatory. As human beings it is our natural desire to form relationships with others and to not be alone. The fourth element is meaning. Meaning refers to what drives a person to work (e.g., children or family) and find purpose in their life. When someone finds meaning in their work they are overall happier. Lastly, the fifth element is accomplishment. People finds strength and self-worth when meeting a milestone and accomplishing a task thus enhancing their well-being. Each element is an independent assessment of well-being that allows and individual to flourish when all elements are fulfilled. Thus, the overall basis of the PERMA theory is to identify that alleviation of suffering does not equate to flourishing. For example, distributing LGBTQ paraphernalia may alleviate the thought of feeling unwelcome (associated with positive emotion), but does not allow LGBTQ trainees to thrive in a department that has unconscious bias towards LGBTQ individuals (associated with engagement, relationships, and accomplishment).

We also embedded an intersectionality framework within the PERMA theory. Intersectionality is a framework to explore how multiple parts of one’s identity (e.g., sexuality, race, gender) interact to impact systems. Thus in order to focus on and improve LGBTQ well-being, we must understand how the current training environment impacts their ability to flourish through an intersectionality lens. Improving our understanding of the current lived experiences of LGBTQ trainees in academic institutions can guide targeted interventions focused on helping trainees flourish in their environments for the overall academic and clinical success of these trainees as well as the patients they serve.

Thus, the objective of this exploratory study is to compare LGBTQ trainees’ experiences to non-LGBTQ trainees’ experiences within the medical workplace. A secondary objective was to identify if there were any racial differences between trainees’ experiences within the medical workspace. Lastly, a tertiary objective was to investigate if there were differences in experiences based on intersectionality of trainees (i.e., trainees who identify as LGBTQ and a minority) versus those who were LGBTQ and White.

## Methods

### Study design and participants

We conducted a cross-sectional, exploratory study of data collected from GME trainees (residents and fellows) at the largest academic training center located in Birmingham, AL. This self-administered survey was distributed to all GME trainees from 1 December 2020 to 14 January 2021. Inclusion criteria was classification as a university GME resident or fellow during the time the survey was dispersed. Exclusion criterion was any trainee who did not identify their sexual orientation in the survey. Participants were recruited through email communication through the GME listserv. This study was approved by the Institutional Review Board at the University of Alabama at Birmingham.

### Integration of the PERMA theory

**Figure 1.**
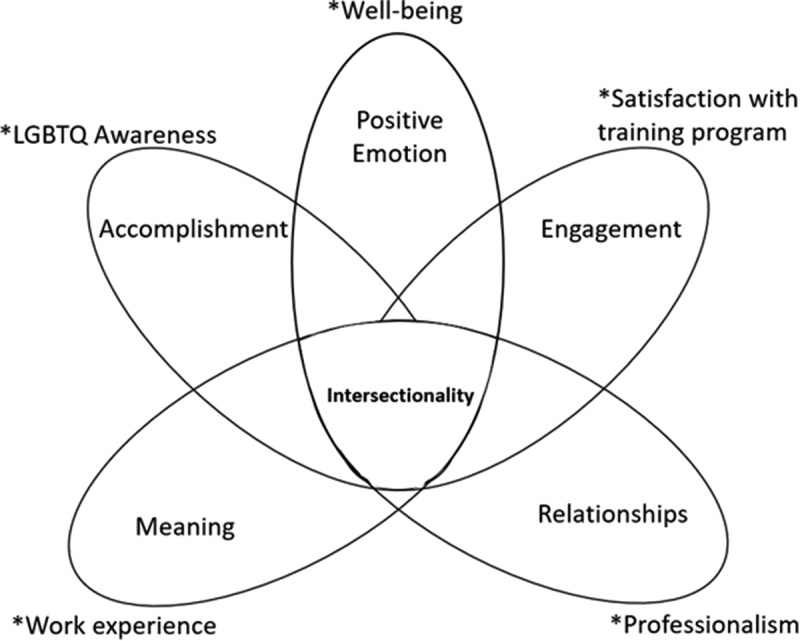
Integration of PERMA theory of well-being with intersectionalities mapped to GME trainee survey sections.

The PERMA theory and intersectionality are the principal theories for this research. Each of the five components of the PERMA theory (Positive emotion, Engagement, Relationships, Meaning, and Accomplishment) are measured independently and should be explored separately. Our survey attempts to explore these elements individually. The scores in any of these elements provides insight regarding the degree of well-being, or flourishing, trainees may have at the institution. In addition, we believe that intersectionality, especially in LGBTQ individuals, provides another layer of inhibition for individuals to flourish when combatted with unsupportive environments and should be included in the evaluation using this theory (Figure 1).

* = Corresponding survey sections

### Survey design

An 80-item survey, deployed annually since 2015 to assess the work and learning environment among trainees, was modified to include an additional section labelled ‘LGBTQ awareness’. The new survey was broken down into six sections: background information, professionalism, well-being, work experience, satisfaction with training program, and LGBTQ awareness. Demographic variables (age, postgraduate year (PGY) level, Accreditation Council of Graduate Medical Education (ACGME) category, race, and relationship status) were collected in the section entitled ‘background information’ to assess for similarities between both groups. The section pertaining to LGBTQ awareness consisted of seven questions (Addendum A) that were generated based on feedback from an internal institutional review committee focused on LGBTQ quality and prior studies with similar assessments [11,12]. In conjunction with the PERMA theory, we equated professionalism to relationships, well-being to positive emotion, work experience to meaning, satisfaction with training program to engagement, and LGBTQ awareness to accomplishment. LGBTQ individuals were defined based on sexual orientation. All individuals who identified as transsexual regarding their gender identity were included in LGBTQ. Based on these responses individuals were stratified into two categories: Straight and LGBTQ. The estimated completion time was approximately 10 minutes. There was no compensation for completion of the survey. All data was collected anonymously and stored in a REDCap database.

### Statistical analysis

Descriptive statistics were used to summarize patient characteristics. Contingency tables and Fisher’s exact test were performed to identify outcomes associated with sexual orientation and gender identity particularly regarding professionalism, well-being, and satisfaction with training. Data reported in this study reflected statistical significance when *p* < 0.05. Given the small cell size in primary analysis of our secondary and tertiary objectives, we ran a separate analysis with collapsed variables. All outcomes were collapsed to the following: agree (slightly agree, moderately agree, and strongly agree); disagree (slightly disagree, moderately disagree, strongly disagree, or neutral); No (never); Yes (once, occasionally, and frequently); important (very important, moderately important, and somewhat important); and not important (less important, not important at all, and I’m not sure). All analyses were performed using R software, version 4.0.2 (R Core Team, 2021).

## Results

Eight hundred and forty GME trainees took the survey. In total, 730 trainees met inclusion criteria with 707 (96%) Straight trainees and 23 (3.2%) LGBTQ trainees (Figure 1). There were no significant differences between the two groups based on age, postgraduate year (PGY) level, American College of Graduate Medical Education (ACGME) category, and relationship status. There were differences in gender (p = <0.01) and race (p = 0.01) between the two groups with the Straight cohort containing more White, cis-gendered males (52% vs 72%) (Table 1). We did not include these characteristics as confounders in our analysis given the small sample size, the inherent bias based in the cohorts, and the overall similarity within the general medical population.

**Table 1.** Baseline demographics.

Characteristics		N	LGBTQ N(%)	Straight N(%)	P value
Age	18–24	3	1(4.3)	2(0.3)	
	25–34	722	20(87)	631(89.3)	
	35+	87	2(8.7)	72(10.2)	
	NA*	28	0(0)	2(0.3)	0.11
PGY level	PGY1	135	5(21.7)	115(16.3)	
	PGY2	146	5(21.7)	132(18.7)	
	PGY3	172	3(13)	148(20.9)	
	PGY4	157	8(34.8)	126(17.8)	
	PGY5	105	1(4.3)	98(13.9)	
	PGY6	67	0(0)	61(8.6)	
	PGY7 or higher	28	1(4.3)	24(3.4)	
	NA*	30	0(0)	3(0.4)	0.21
ACGME Category	Dentistry	17	0(0)	11(1.6)	
	Hospital-based	195	4(17.4)	176(24.9)	
	Medical	427	11(47.8)	353(49.9)	
	Surgical	201	8(34.8)	167(23.6)	
	NA*	0	0(0)	0(0)	0.64
Gender	Male	462	11(47.8)	402(56.9)	
	Female	342	10(43.5)	299(42.3)	
	Transgender (male to female)	1	1(4.3)	0(0)	
	Transgender (female to male)	1	1(4.3)	0(0)	
	Non-binary	1	0(0)	0(0)	
	Not listed	4	0(0)	3(0.4)	
	NA*	29	0(0)	3(0.4)	<0.01
Race	White	523	12(52.5)	509(72)	
	Black/African-American/African	32	4(17.4)	27(3.8)	
	Asian	109	3(13)	103(14.6)	
	Other	71	4(17.4)	64(9.1)	
	NA*	105	0(0)	4(0.6)	0.01
Relationship status	Never married	297	12(52.2)	255(36.1)	
	Married or Domestic partnership	492	11(47.8)	434(61.4)	
	Widowed	1	0(0)	1(0.1)	
	Divorced	13	0(0)	13(1.8)	
	Separated	4	0(0)	3(0.4)	
	NA*	33	0(0)	1(0.1)	0.41

LGBTQ = Lesbian, Gay, Bisexual, Transgender, and Questioning; NA = Not answered; PGY = postgraduate year; ACGME = Accreditation Council of General Medical Education

*Not included in the calculation of the p-value from the Fisher’s exact test.

Analysis of the LGBTQ-specific questions of the survey revealed that the majority of individuals identified as Straight (707 (84%)), Gay (10 (1%)), and Bisexual (8 (1%)) and preferred either she/her (309 (37%)) or he/him (409 (49%)) pronouns (Table 2). Only 0.1% of trainees reported their department being aware of their gender identity. A majority of individuals agreed that the institution (551 (66%)) and department (589 (70%)) have a welcoming LGBTQ environment. When looking specifically at LGBTQ respondents, 60.8% (n = 14) agreed that the institution had a welcoming environment and 78.3% (n = 18) agreed that their department was welcoming. Lastly, only 0.1% of trainees responded to being willing to mentor someone who identifies as LGBTQ. All respondents willing to mentor LGBTQ individuals identified as LGBTQ (data not shown). However, 99.5% of individuals did not answer this question. The low response to the question regarding mentorship may be due to survey fatigue as this was one of the last questions of the survey.

**Table 2.** LGBTQ-specific questions.

Questions		N(%)
In terms of sexual orientation, what do you think of yourself as (please check all that apply)?	Straight	707(84.2)
	Gay	10(1.2)
	Lesbian	1(0.1)
	Bisexual	8(1)
	Ques	1(0.1)
	Asexual	0(0)
	Pansexual	3(0.4)
	NA	110(13.1)
What are your gender pronouns?	She/her	309(36.8)
	He/him	409(48.7)
	They/them	0(0)
	Not listed above	7(0.8)
	NA	115(13.7)
Is your department aware of your gender identity?	Yes	1(0.1)
	No	1(0.1)
	NA	838(99.8)
The institution has a welcoming LGBTQ+ environment.	Strongly Agree	233(27.7)
	Agree	318(37.9)
	Neutral	175(20.8)
	Disagree	6(0.7)
	Strongly Disagree	0(0)
	NA	108(12.9)
My department has a welcoming LGBTQ+ environment.	Strongly Agree	287(34.2)
	Agree	302(36)
	Neutral	138(16.4)
	Disagree	4(0.5)
	Strongly Disagree	0(0)
	NA	109(13.0)
Would you be willing to mentor someone who identifies as LGBTQ+?	Yes	1(0.1)
	No	3(0.4)
	NA	836(99.5)

LGBTQ = Lesbian, Gay, Bisexual, Transgender, and Questioning; NA = Not answered

Table 3 reveals findings pertaining to professionalism, well-being, and satisfaction with training programs. Significant differences in experiences with professionalism were found. LGBTQ trainees were more likely to report being subjected to racially or ethnically offensive remarks/names (p = 0.03) and being subjected to offensive remarks/names related to sexual orientation (p = 0.01). There were no other significant differences seen pertaining to professionalism between the two groups. There was no evidence of difference based on well-being or satisfaction with the training program between LGBTQ and Straight trainees.

**Table 3.** Analysis of responses to questions regarding professionalism, well-being, and satisfaction with training program between Lesbian, Gay, Bisexual, Transgender, and Questioning (LGBTQ) and Straight trainees.

		N	LGBTQ N(%)	Straight N(%)	P value
Professionalism					
Been publicly humiliated?	Never	691	23(100)	624(88.3)	
	Once	46	0(0)	44(6.2)	
	Occasionally	43	0(0)	31(4.4)	
	Frequently	5	0(0)	4(0.6)	
	NA*	55	0(0)	4(0.6)	0.53
Been threatened with physical harm?	Never	763	23(100)	684(96.7)	
	Once	12	0(0)	12(1.7)	
	Occasionally	8	0(0)	6(0.8)	
	Frequently*	0	0(0)	0(0)	
	NA*	57	0(0)	5(0.7)	>0.99
Been physically harmed?	Never	778	23(100)	697(98.6)	
	Once	6	0(0)	5(0.7)	
	Occasionally*	0	0(0)	0(0)	
	Frequently*	0	0(0)	0(0)	
	NA*	56	0(0)	5(0.7)	>0.99
Been subjected to unwanted sexual advances?	Never	758	22(95.7)	680(96.2)	
	Once	15	0(0)	13(1.8)	
	Occasionally	11	1(4.3)	9(1.3)	
	Frequently*	0	0(0)	0(0)	
	NA*	56	0(0)	5(0.7)	0.32
Been subjected to offensive sexist remarks/names?	Never	712	21(91.3)	643(90.9)	
	Once	26	0(0)	23(3.3)	
	Occasionally	43	2(8.7)	34(4.8)	
	Frequently	3	0(0)	2(0.3)	
	NA*	56	0(0)	5(0.7)	0.47
Been denied opportunities for training or rewards based on gender?	Never	756	22(95.7)	681(96.3)	
	Once	12	0(0)	11(1.6)	
	Occasionally	13	1(4.3)	8(1.1)	
	Frequently	3	0(0)	2(0.3)	
	NA*	56	0(0)	5(0.7)	0.33
Been subjected racially or ethnically offensive remarks/names?	Never	718	19(82.6)	648(91.7)	
	Once	30	0(0)	27(3.8)	
	Occasionally	33	4(17.4)	25(3.5)	
	Frequently	1	0(0)	1(0.1)	
	NA*	58	0(0)	6(0.8)	0.03
Been denies opportunities for training or rewards based on race or ethnicity?	Never	766	22(95.7)	687(97.2)	
	Once	7	0(0)	6(0.8)	
	Occasionally	6	0(0)	6(0.8)	
	Frequently	1	0(0)	1(0.1)	
	NA*	60	1(4.3)	7(1.0)	>0.99
Been subjected to offensive remarks/names related to sexual orientation?	Never	775	21(91.3)	698(98.7)	
	Once	4	1(4.3)	3(0.4)	
	Occasionally	3	1(4.3)	1(0.1)	
	Frequently	1	0(0)	0(0)	
	NA*	57	0(0)	5(0.7)	0.01
Been denied opportunities for training or rewards based on sexual orientation?	Never	780	22(95.7)	700(99.0)	
	Once	2	1(4.3)	1(0.1)	
	Occasionally*	0	0(0)	0(0)	
	Frequently*	0	0(0)	0(0)	
	NA*	58	0(0)	6(0.8)	0.06
Shouting, use of belittling or abusive language	Never	632	21(91.3)	577(81.6)	
	Once	63	1(4.3)	59(8.3)	
	Occasionally	70	0(0)	62(8.8)	
	Frequently	6	1(4.3)	4(0.6)	
	NA*	69	0(0)	5(0.7)	0.10
Verbal, written, or physical personal attacks directed at others	Never	705	20(87.0)	649(91.8)	
	Once	27	1(4.3)	22(3.1)	
	Occasionally	36	2(8.7)	29(4.1)	
	Frequently	4	0(0)	3(0.4)	
	NA*	68	0(0)	4(0.6)	0.36
Public derogatory comments about quality of care by other professionals	Never	631	20(87)	582(82.3)	
	Once	34	0(0)	32(4.5)	
	Occasionally	104	3(13)	88(12.4)	
	Frequently	4	0(0)	3(0.4)	
	NA*	67	0(0)	2(0.3)	0.83
Inappropriate expressions of anger (e.g., throwing things, destruction of property)	Never	738	23(100)	673(95.2)	
	Once	18	0(0)	17(2.4)	
	Occasionally	11	0(0)	9(1.3)	
	Frequently	2	0(0)	2(0.3)	
	NA*	71	0(0)	6(0.8)	>0.99
Physical assault	Never	770	23(100)	702(99.3)	
	Once	1	0(0)	1(0.1)	
	Occasionally*	0	0(0)	0(0)	
	Frequently*	0	0(0)	0(0)	
	NA*	69	0(0)	4(0.6)	>0.99
Unwelcome or wanted physical advances of a sexual nature	Never	762	23(100)	694(98.2)	
	Once	4	0(0)	4(0.6)	
	Occasionally	5	0(0)	5(0.7)	
	Frequently*	0	0(0)	0(0)	
	NA*	69	0(0)	4(0.6)	>0.99
Verbal abuse or joking based on gender	Never	712	21(91.3)	655(92.6)	
	Once	20	0(0)	18(2.5)	
	Occasionally	36	1(4.3)	28(4.0)	
	Frequently	1	0(0)	1(0.1)	
	NA*	71	1(4.3)	5(0.7)	0.78
Verbal abuse or joking based on race/ethnicity	Never	735	20(87.0)	673(95.2)	
	Once	13	2(8.7)	11(1.6)	
	Occasionally	21	1(4.3)	17(2.4)	
	Frequently	1	0(0)	1(0.1)	
	NA*	70	0(0)	5(0.7)	0.08
Verbal abuse or joking based on sexual orientation	Never	754	23(100)	688(97.3)	
	Once	7	0(0)	7(1.0)	
	Occasionally	9	0(0)	7(1.0)	
	Frequently*	0	0(0)	0(0)	
	NA*	70	0(0)	5(0.7)	>0.99
Well-Being					
Have you felt emotionally drained from your work?	Yes	424	10(43.5)	310(55.9)	
	No	331	13(56.5)	395(43.8)	
	NA*	85	0(0)	2(0.3)	0.29
Have you worried that your work is hardening you emotionally?	Yes	329	8(34.8)	307(43.4)	
	No	428	15(65.2)	400(56.6)	
	NA*	83	0(0)	0(0)	0.52
Have you often been bothered by feeling down, depressed, or hopeless?	Yes	189	6(26.1)	175(24.8)	
	No	565	17(73.9)	529(74.8)	
	NA*	86	0(0)	3(0.4)	0.81
Have you fallen asleep while sitting inactive in a public place?	Yes	138	2(8.7)	132(18.7)	
	No	618	21(91.3)	574(81.2)	
	NA*	84	0(0)	1(0.1)	0.28
Have you felt that all things you had to do were piling up so high that you could not overcome them?	Yes	210	10(43.5)	192(27.2)	
	No	545	13(56.5)	514(72.7)	
	NA*	85	0(0)	1(0.1)	0.10
Have you been bothered by emotional problems (such as feeling anxious, depressed, or irritable)?	Yes	312	10(43.5)	294(41.6)	
	No	442	13(56.5)	410(58.0)	
	NA*	86	0(0)	3(0.4)	>0.99
Has your physical health interfered with your ability to do your daily work at home and/or away from home?	Yes	78	2(8.7)	72(10.2)	
	No	676	21(91.3)	632(89.4)	
	NA*	86	0(0)	3(0.4)	>0.99
During the past month, have you often been bothered by little interest or pleasure in doing things?	Yes	136	4(17.4)	124(17.5)	
	No	619	19(82.6)	581(82.2)	
	NA*	85	0(0)	2(0.3)	>0.99
The work I do is meaningful to me.	Very strongly disagree	17	1(4.3)	16(2.3)	
	Strongly disagree	9	1(4.3)	7(1.0)	
	Disagree	3	0(0)	3(0.4)	
	Neutral	34	1(4.3)	31(4.4)	
	Agree	194	7(30.4)	175(24.8)	
	Strongly agree	298	7(30.4)	283(40.0)	
	Very Strongly agree	201	6(26.1)	191(27.0)	
	NA*	84	0(0)	1(0.1)	0.41
How important is your mental health and wellness to your program?	Very important	373	14(60.9)	350(49.5)	
	Moderately important	214	4(17.4)	204(28.9)	
	Somewhat important	113	4(17.4)	104(14.7)	
	Less important	23	0(0)	20(2.8)	
	Not important at all	15	1(4.3)	12(1.7)	
	I’m not sure	18	0(0)	17(2.4)	
	NA*	84	0(0)	0(0)	0.54
Satisfaction with Training Program					
Are you satisfied with the quality of training you are receiving?	Yes	709	20(87)	677(95.8)	
	No	32	3(13)	28(4.0)	
	NA*	99	0(0)	2(0.3)	0.07
I would recommend my training program to others.	Strongly disagree	19	0(0)	17(2.4)	
	Moderately disagree	10	0(0)	9(1.3)	
	Slightly disagree	14	0(0)	14(2.0)	
	Slightly agree	62	3(13.0)	58(8.2)	
	Moderately agree	149	3(13.0)	143(20.2)	
	Strongly agree	489	17(73.9)	466(65.9)	
	NA*	97	0(0)	0(0)	0.83

LGBTQ = Lesbian, Gay, Bisexual, Transgender, and Questioning; NA = Not answered

*Not included in the calculation of the p-value from the Fisher’s exact test.

Survey responses based on race were obtained (Table 4). Regarding professionalism, we found a difference between racial groups in reports of being denied opportunities for training or rewards based on gender (2.3% White, 9.3% Black, 2.7% Asian, 5.6% Other; p = 0.02) and race (0.6% White, 3.1% Black, 3.6% Asian, 7% Other; p < 0.01), and witnessing verbal abuse or joking (2.9% White, 9.3% Black, 6.4% Asian, 8.4% Other; p = 0.01). Regarding satisfaction with their quality of training based on race, trainees self-identifying as Other were more likely to report dissatisfaction with their quality of training (2.7% White, 6.2% Black, 4.6% Asian, 16% Other; p < 0.01). In regard to well-being, no evidence of difference was identified based on race. After collapsing variables to bimodal responses, we found statistical significance Other respondents were more likely to reporting being publicly humiliated (9.6% White, 3.1% Black, 10.1% Asian, 21.1% Other; p = 0.02) and were less likely to report an importance of mental health and wellness to their program (94.6% White, 93.7% Black, 89.9% Asian, 84.5% Other; p = 0.01). Asians and Other trainees were more likely to report being subjected to racially or ethnically offensive remarks/names (3.4% White, 9.3% Black, 20.2% Asian, 16.9% Other; p < 0.01). Black trainees were less likely to recommend their training program to others (96% White, 87.5% Black, 90.6% Asian, 90.1% Other; p = 0.01). We did not find any further significance in respondents reporting being denied opportunities for training or rewards based on gender (p = 0.06). We found no evidence of differences in experiences among LGBTQ trainees based on race (data not shown).

**Table 4.** Analysis of responses to questions regarding professionalism, well-being, and satisfaction with training program based on race.

		N	White N(%)	Black N(%)	Asian N(%)	Other N(%)	P value
Professionalism							
Been publicly humiliated?	Never	691	470(89.9)	31(96.9)	97(89.0)	56(78.9)	
	Once	46	34(6.5)	0(0)	5(4.6)	5(7.0)	
	Occasionally	43	15(2.9)	1(3.1)	4(3.7)	9(12.7)	
	Frequently	5	1(0.2)	0(0)	2(1.8)	1(1.4)	
	NA*	55	3(0.6)	0(0)	1(0.9)	0(0)	N/A
Been threatened with physical harm?	Never	763	507(96.9)	32(100)	105(96.3)	68(95.8)	
	Once	12	8(1.5)	0(0)	3(2.8)	1(1.4)	
	Occasionally	8	4(0.8)	0(0)	0(0)	2(2.8)	
	Frequently*	0	0(0)	0(0)	0(0)	0(0)	
	NA*	57	4(0.8)	0(0)	1(0.9)	0(0)	0.46
Been physically harmed?	Never	778	515(98.5)	32(100)	107(98.2)	71(100)	
	Once	6	4(0.8)	0(0)	1(0.9)	0(0)	
	Occasionally*	0	0(0)	0(0)	0(0)	0(0)	
	Frequently*	0	0(0)	0(0)	0(0)	0(0)	
	NA*	56	4(0.8)	0(0)	1(0.9)	0(0)	>0.99
Been subjected to unwanted sexual advances?	Never	758	504(96.4)	31(96.9)	105(96.3)	68(95.8)	
	Once	15	9(1.7)	0(0)	2(1.8)	1(1.4)	
	Occasionally	11	6(1.1)	1(3.1)	1(0.9)	2(2.8)	
	Frequently*	0	0(0)	0(0)	0(0)	0(0)	
	NA*	56	4(0.8)	0(0)	1(0.9)	0(0)	0.67
Been subjected to offensive sexist remarks/names?	Never	712	479(91.6)	30(93.8)	98(89.9)	62(87.3)	
	Once	26	14(2.7)	1(3.1)	4(3.7)	3(4.2)	
	Occasionally	43	25(4.8)	0(0)	6(5.5)	6(8.5)	
	Frequently	3	2(0.4)	0(0)	0(0)	0(0)	
	NA*	56	3(0.6)	1(3.1)	1(0.9)	0(0)	N/A
Been denied opportunities for training or rewards based on gender?	Never	756	507(96.9)	29(90.6)	105(96.3)	67(94.4)	
	Once	12	8(1.5)	2(6.2)	0(0)	1(1.4)	
	Occasionally	13	3(0.6)	1(3.1)	2(1.8)	3(4.2)	
	Frequently	3	1(0.2)	0(0)	1(0.9)	0(0)	
	NA*	56	4(0.8)	0(0)	1(0.9)	0(0)	0.02
Been subjected racially or ethnically offensive remarks/names?	Never	718	500(95.6)	29(90.6)	86(78.9)	59(83.1)	
	Once	30	8(1.5)	1(3.1)	12(11.0)	5(7.0)	
	Occasionally	33	10(1.9)	2(6.2)	9(8.3)	7(9.9)	
	Frequently	1	0(0)	0(0)	1(0.9)	0(0)	
	NA*	58	5(1.0)	0(0)	1(0.9)	0(0)	N/A
Been denies opportunities for training or rewards based on race or ethnicity?	Never	766	515(98.5)	29(90.6)	104(95.4)	66(93.0)	
	Once	7	3(0.6)	0(0)	1(0.9)	2(2.8)	
	Occasionally	6	0(0)	1(3.1)	2(1.8)	3(4.2)	
	Frequently	1	0(0)	0(0)	1(0.9)	0(0)	
	NA*	60	5(1.0)	2(6.2)	1(0.9)	0(0)	<0.01
Been subjected to offensive remarks/names related to sexual orientation?	Never	775	517(98.9)	31(96.9)	107(98.2)	70(98.6)	
	Once	4	2(0.4)	0(0)	0(0)	1(1.4)	
	Occasionally	3	1(0.2)	0(0)	1(0.9)	0(0)	
	Frequently	1	0(0)	0(0)	0(0)	0(0)	
	NA*	57	3(0.6)	1(3.1)	1(0.9)	0(0)	0.42
Been denied opportunities for training or rewards based on sexual orientation?	Never	780	518(99.0)	31(96.9)	107(98.2)	71(100)	
	Once	2	1(0.2)	0(0)	1(0.9)	0(0)	
	Occasionally*	0	0(0)	0(0)	0(0)	0(0)	
	Frequently*	0	0(0)	0(0)	0(0)	0(0)	
	NA*	58	4(0.8)	1(3.1)	1(0.9)	0(0)	0.49
Shouting, use of belittling or abusive language	Never	632	429(82.0)	29(90.6)	90(82.6)	56(78.9)	
	Once	63	46(8.8)	2(6.2)	8(7.3)	4(5.6)	
	Occasionally	70	44(8.4)	1(3.1)	8(7.3)	7(9.9)	
	Frequently	6	1(0.2)	0(0)	2(1.8)	2(2.8)	
	NA*	69	3(0.6)	0(0)	1(0.9)	2(2.8)	N/A
Verbal, written, or physical personal attacks directed at others	Never	705	485(92.7)	29(90.6)	99(90.8)	62(87.3)	
	Once	27	16(3.1)	2(6.2)	4(3.7)	1(1.4)	
	Occasionally	36	20(3.8)	1(3.1)	4(3.7)	5(7.0)	
	Frequently	4	0(0)	0(0)	1(0.9)	2(2.8)	
	NA*	68	2(0.4)	0(0)	1(0.9)	1(1.4)	N/A
Public derogatory comments about quality of care by other professionals	Never	631	433(82.8)	29(90.6)	90(82.6)	56(78.9)	
	Once	34	20(3.8)	0(0)	7(6.4)	5(7.0)	
	Occasionally	104	68(13.0)	3(9.4)	10(9.2)	8(11.3)	
	Frequently	4	1(0.2)	0(0)	1(0.9)	1(1.4)	
	NA*	67	1(0.2)	0(0)	1(0.9)	1(1.4)	N/A
Inappropriate expressions of anger (e.g., throwing things, destruction of property)	Never	738	501(95.8)	31(96.9)	103(94.5)	66(93.0)	
	Once	18	11(2.1)	1(3.1)	2(1.8)	2(2.8)	
	Occasionally	11	6(1.1)	0(0)	2(1.8)	1(1.4)	
	Frequently	2	0(0)	0(0)	1(0.9)	1(1.4)	
	NA*	71	5(1.0)	0(0)	1(0.9)	1(1.4)	0.32
Physical assault	Never	770	521(99.6)	31(96.9)	108(99.1)	69(97.2)	
	Once	1	0(0)	0(0)	0(0)	1(1.4)	
	Occasionally*	0	0(0)	0(0)	0(0)	0(0)	
	Frequently*	0	0(0)	0(0)	0(0)	0(0)	
	NA*	69	2(0.4)	1(3.1)	1(0.9)	1(1.4)	0.14
Unwelcome or wanted physical advances of a sexual nature	Never	762	516(98.7)	31(96.9)	106(97.2)	68(95.8)	
	Once	4	2(0.4)	0(0)	1(0.9)	1(1.4)	
	Occasionally	5	2(0.4)	1(3.1)	1(0.9)	1(1.4)	
	Frequently*	0	0(0)	0(0)	0(0)	0(0)	
	NA*	69	3(0.6)	0(0)	1(0.9)	1(1.4)	0.14
Verbal abuse or joking based on gender	Never	712	488(93.3)	29(90.6)	103(94.5)	60(84.5)	
	Once	20	12(2.3)	1(3.1)	3(2.8)	3(4.2)	
	Occasionally	36	20(3.8)	2(6.2)	1(0.9)	5(7.0)	
	Frequently	1	0(0)	0(0)	1(0.9)	0(0)	
	NA*	71	3(0.6)	0(0)	1(0.9)	3(4.2)	0.21
Verbal abuse or joking based on race/ethnicity	Never	735	505(96.6)	29(90.6)	101(92.7)	63(88.7)	
	Once	13	5(1.0)	1(3.1)	5(4.6)	2(2.8)	
	Occasionally	21	10(1.9)	2(6.2)	1(0.9)	4(5.6)	
	Frequently	1	0(0)	0(0)	1(0.9)	0(0)	
	NA*	70	3(0.6)	0(0)	1(0.9)	2(2.8)	0.01
Verbal abuse or joking based on sexual orientation	Never	754	511(97.7)	32(100)	105(96.3)	68(95.8)	
	Once	7	4(0.8)	0(0)	3(2.8)	0(0)	
	Occasionally	9	5(1.0)	0(0)	0(0)	1(1.4)	
	Frequently*	0	0(0)	0(0)	0(0)	0(0)	
	NA*	70	3(0.6)	0(0)	1(0.9)	2(2.8)	0.41
Well-Being							
Have you felt emotionally drained from your work?	Yes	424	306(58.5)	15(46.9)	52(47.7)	37(52.1)	
	No	331	216(41.3)	17(53.1)	56(51.4)	34(47.9)	
	NA*	85	1(0.2)	0(0)	1(0.9)	0(0)	0.13
Have you worried that your work is hardening you emotionally?	Yes	329	238(45.5)	10(31.2)	41(37.6)	31(43.7)	
	No	428	285(54.5)	22(68.8)	68(62.4)	40(56.3)	
	NA*	83	0(0)	0(0)	0(0)	0(0)	0.23
Have you often been bothered by feeling down, depressed, or hopeless?	Yes	189	127(24.3)	9(28.1)	28(25.7)	19(26.8)	
	No	565	395(75.5)	23(71.9)	80(73.4)	51(71.8)	
	NA*	86	1(0.2)	0(0)	1(0.9)	1(1.4)	0.88
Have you fallen asleep while sitting inactive in a public place?	Yes	138	94(18)	5(15.6)	17(15.6)	17(23.9)	
	No	618	429(82)	27(84.4)	91(83.5)	54(76.1)	
	NA*	84	0(0)	0(0)	1(0.9)	0(0)	0.55
Have you felt that all things you had to do were piling up so high that you could not overcome them?	Yes	210	147(28.1)	10(31.2)	31(28.4)	18(25.4)	
	No	545	376(71.9)	22(68.8)	77(70.6)	52(73.2)	
	NA*	85	0(0)	0(0)	1(0.9)	1(1.4)	0.94
Have you been bothered by emotional problems (such as feeling anxious, depressed, or irritable)?	Yes	312	214(40.9)	13(40.6)	45(41.3)	34(47.9)	
	No	442	306(58.5)	19(59.4)	64(58.7)	37(52.1)	
	NA*	86	3(0.6)	0(0)	0(0)	0(0)	0.75
Has your physical health interfered with your ability to do your daily work at home and/or away from home?	Yes	78	48(9.2)	5(15.6)	11(10.1)	10(14.1)	
	No	676	473(90.4)	27(84.4)	98(89.9)	60(84.5)	
	NA*	86	2(0.4)	0(0)	0(0)	1(1.4)	0.34
During the past month, have you often been bothered by little interest or pleasure in doing things?	Yes	136	94(18.0)	7(21.9)	15(13.8)	14(19.7)	
	No	619	428(81.8)	25(78.1)	94(86.4)	56(78.9)	
	NA*	85	1(0.2)	0(0)	0(0)	1(1.4)	0.57
The work I do is meaningful to me.	Very strongly disagree	17	9(1.7)	1(3.1)	5(4.6)	2(2.8)	
	Strongly disagree	9	5(1.0)	1(3.1)	2(1.8)	0(0)	
	Disagree	3	2(0.4)	0(0)	0(0)	1(1.4)	
	Neutral	34	22(4.2)	0(0)	6(5.5)	5(7.0)	
	Agree	194	141(27.0)	4(12.5)	27(24.8)	14(19.7)	
	Strongly Agree	298	209(40.0)	15(46.9)	44(40.4)	23(32.4)	
	Very Strongly Agree	201	135(25.8)	11(34.4)	24(22.0)	26(36.6)	
	NA*	84	0(0)	0(0)	1(0.9)	0(0)	N/A
How important is your mental health and wellness to your program?	Very important	373	258(49.3)	18(56.2)	50(45.9)	39(54.9)	
	Moderately important	214	160(30.6)	7(21.9)	30(27.5)	13(18.3)	
	Somewhat important	113	77(14.7)	5(15.6)	18(16.5)	8(11.3)	
	Less important	23	12(2.3)	0(0)	3(2.8)	6(8.5)	
	Not important at all	15	5(1.0)	1(3.1)	4(3.7)	4(5.6)	
	I’m not sure	18	11(2.1)	1(3.1)	4(3.7)	1(1.4)	
	NA*	84	0(0)	0(0)	0(0)	0(0)	N/A
Satisfaction with Training Program							
Are you satisfied with the quality of training you are receiving?	Yes	709	507(96.9)	30(93.8)	104(95.4)	60(84.5)	
	No	32	14(2.7)	2(6.2)	5(4.6)	11(15.5)	
	NA*	99	2(0.4)	0(0)	0(0)	0(0)	<0.01
I would recommend my training program to others.	Strongly disagree	19	9(1.7)	0(0)	7(6.4)	2(2.8)	
	Moderately disagree	10	4(0.8)	3(9.4)	2(1.8)	1(1.4)	
	Slightly disagree	14	8(1.5)	1(3.1)	1(0.9)	4(5.6)	
	Slightly agree	62	34(6.5)	3(9.4)	12(11.0)	11(15.5)	
	Moderately agree	149	103(19.7)	4(12.5)	26(23.6)	15(21.1)	
	Strongly agree	489	365(69.8)	21(65.6)	61(56.0)	38(53.5)	
	NA*	97	0(0)	0(0)	0(0)	0(0)	N/A

NA = Not answered

*Not included in the calculation of the p-value from the Fisher’s exact test.

## Discussion

This is the first study to assess the training experiences of LGBTQ trainees in a southern training program utilizing a PERMA theory lens. Our study demonstrates three percent of trainees identify as LGBTQ, which is consistent with the percent of LGBTQ residents in Alabama and the country as a whole [3,8]. We also identified variability in experiences regarding professionalism/relationships between LGBTQ and Straight trainees at a single, large teaching institution, with some of the variability explained by intersectionalities such as race.

### Comparison with prior studies

This study is one of few prior studies that assess the experiences among LGBTQ trainees. To our knowledge, this is the only study that solely compared LGBTQ and Straight residents and fellows. Prior studies have evaluated LGBTQ medical students’ and residents’ experiences and have revealed similar findings seen in our analysis. Sanchez, et al., found that approximately 40% of LGBTQ physicians and trainees avoided disclosure of their sexual orientation due to fear of harassment, discrimination, or negative impact[12]. Dimant, et al., found that transgender and gender non-binary (TGNB) medical students and physicians were more likely to report no barriers in the work environment but reported hearing or witnessing derogatory comments about TGNB individuals[11]. In contrast, Lapinski and Sexton found in an analysis of 4112 medical students at six US osteopathic medical schools that LGB students were more likely to be depressed than heterosexual/Straight students, indicate lower levels of perceived social support, and were more likely to feel discomfort with disclosure of their sexual orientation[13]. Also, a study by Anderson et al. found that microaggressions occur frequently within medical school training and result in positive depression screening and decreased medical school training satisfaction[14]. Although the studies were variable in design, there were some common themes shared with our study: assessment of well-being, fear of disclosure of identity, and barriers within the workplace for LGBTQ trainees. Consistent with the PERMA theory shows, ‘suffering’ during training as an LGBTQ individual is not uncommon and impacts trainees’ ability to flourish in the workplace.

### Assessment of well-being (positive emotion & meaning)

Our study found no evidence of differences in well-being/positive emotion between LGBTQ and Straight trainees, even in sub-analyses based on racial differences. In addition, over 60% of all respondents felt that the institution and their respective department were LGBTQ friendly. Understanding LGBTQ trainees’ perspectives of their department’s environment is important because literature has found LGBTQ individuals are at an increased risk for problems with mental health (e.g., depression, suicide, psychosocial disorders) [15–21]. Also, having a supportive environment fosters more social interaction and allows LGBTQ individuals the ability to create bonds and potential mentorship [22,23]. Thereby explaining the link between positive emotion, relationships, and engagement and their impact on flourishing.

### Fear of disclosure (relationships)

The stigma that is associated with ‘The South’ in regard to acceptance of LGBTQ lifestyle still plagues the minds of trainees considering moving to these areas. The fear of disclosure of sexual orientation is a common concern among LGBTQ trainees and interferes with the ability to form relationships. Prior studies identify that LGBTQ trainees do not desire to disclose their sexual orientation due to fear of harassment, discrimination, and missed career opportunities [5,24–27]. We did not identify any differences between LGBTQ and Straight trainees pertaining to being denied opportunities based on sexual orientation (p = 0.06). However, individuals who identify as both racially and ethnically underrepresented in medicine and LGBTQ may feel very uncomfortable with disclosing their sexual orientation given additional biases compounded by race.

### Barriers in the workplace (engagement & accomplishment)

Lastly, although institutions are beginning to identify barriers to LGBTQ trainees’ experience in the workplace, interventions and strategies to remove such barriers have rarely been sought out. Aforementioned, most individuals felt the institution and their department were LGBTQ friendly; however, we are unclear if this same perspective is shared by LGBTQ trainees specifically. Eliason, et al. found that LGBTQ physicians reported being denied referrals from heterosexual/Straight colleagues and were denied privileges or promotion based on their sexuality[24]. Some studies have also shown that LGBTQ medical students, trainees, and physicians are likely to witness derogatory comments, substandard care, or refusal of care towards LGBTQ patients, which was also seen in our cohort [24,28]. There is a gap in the literature evaluating systematic approaches to rectify grievances to adoption of evidence based practices or policy change. LGBTQ trainees in this environment may be subjected to internalizing their grievances and have negative social interactions with colleagues and mentors which ultimately increases the occurrence of psychosocial problems in this population. These barriers impede trainees’ abilities to fully engage in their work and achieve a sense of accomplishment, thereby inhibiting their ability to flourish in their environment. This is best illustrated by our study finding only 0.1% of trainees who identified as LGBTQ were willing to mentor LGBTQ individuals, suggesting individuals may not be able to dedicate themselves to something or someone for the greater good. Further qualitative studies are needed.

It is imperative that institutions have a zero-tolerance policy for harassment, discrimination, and inequality and an open-door policy for trainees to report grievances without fear of repercussion.

### Intersectionalities (positive emotion, engagement, and accomplishment)

Our study also suggested intersectionality may be important. Ideally, each component of the PERMA theory is assessed individually; however, the complexity of the effects of intersectionality on individuals may impact their positive emotion, engagement, or accomplishment.

Despite the growing acknowledgment by the AAMC and LCME for a racially diverse workforce, there continues to be significant gaps in making training environments inclusive as is evidenced by 71% of survey respondents being White and 56% identifying as male. Underrepresented in medicine (URiM) trainees are more likely to care for racial and ethnic minorities [29,30], and are also more likely to experience burnout due to implicit and explicit racial biases compared to their White colleagues[31]. Furthermore, according to the most recent 2020 AAMC U.S. Medical School Faculty report, Black and Hispanic physicians are less likely to be in faculty positions compared to Whites and Asians[32]. Although, LGBTQ trainees’ experiences did not vary based on race secondary to low rates of self-reported identification as LGBTQ, understanding how LGBTQ and race intersect among trainees is crucial. One study by Lett et al found that non-cisgender Black individuals are more likely to experience severe mental illness and longer periods of being physically or mentally unwell compared to cisgender Blacks; and had worse self-reported health compared to cisgender Blacks and non-cisgender Whites[33]. These experiences, whether positive or negative, can be compounded in individuals who identify with multiple underrepresented minority groups in medicine (e.g., Black and Gay, Native American and Lesbian, Latino and Transgender, etc.)[28].

### Clinical significance

This institution’s recognition as a HEI Leader is a step in the right direction towards intentionality in creating a LGBTQ friendly environment and is used as a recruitment tool for LGBTQ trainees. However, additional effort needs to be placed on strategies focused on mitigating some of the inequities experienced by LGBTQ trainees at this institution. LGBTQ trainees are more likely to focus on LGBTQ patient care, advocate for LGBTQ issues, mentor LGBTQ trainees, and initiate research into LGBTQ health disparities [12,24]. The lack of support for LGBTQ trainees usually results in trainees leaving an institution which subsequently results in a heteronormative environment which could be detrimental to LGBTQ patient healthcare quality[27].

### Limitations

This study should be viewed within the context of some limitations. Our cohort is from a single, academic institution within the Southeast and cannot be generalized to other academic centers in other geographic locations. We also have a small cohort of LGBTQ trainees, which limits the statistical testing of differences seen within baseline demographics. In addition, a majority of our cohort are White, which limited the ability to assess intersectionalities based on race. However, we believe our baseline demographics are similar to other large, academic institutions, and in that regard, can be generalizable. Recall bias also may have influenced our findings due to the nature of the study. In addition, we excluded 110 (13%) responders due to lack of response to sexual orientation. We found that as the survey progressed there was a higher number of nonresponses, which we believe, is due to survey fatigue. However, given LGBTQ specific questions were the last section of the survey, we believe exclusion of these individuals did not significantly impact our primary outcome.

### Future studies

Future studies are needed to assess the LGBTQ experience at other large academic training institutions to identify common trends to guide universal changes in GME and provide optimal training environments for all individuals regardless of sexual orientation or background.

## Conclusion

Expanding our knowledge of the current LGBTQ and URiM trainee workforce is imperative. This study is the first to contrast the dynamics of GME training between LGBTQ and Straight trainees and highlight the disparities seen with underlying principle of the PERMA theory. Increasing diversity and inclusivity will not only alleviate suffering but allow LGBTQ trainees to flourish and thus improve health outcomes for LGBTQ Alabama residents. It is crucial to continue supporting the advancement of this field of study in order to retain and recruit diverse physicians who are interested in improving the quality of LGBTQ healthcare.
